# Comparison of pretherapeutic osseous tumor volume and standard hematology for prediction of hematotoxicity after PSMA-targeted radioligand therapy

**DOI:** 10.1007/s00259-021-05412-1

**Published:** 2021-05-27

**Authors:** Liam Widjaja, Rudolf A. Werner, Tobias L. Ross, Frank M. Bengel, Thorsten Derlin

**Affiliations:** Department of Nuclear Medicine, Hannover Medical School, Hannover, Germany

**Keywords:** PSMA, Prostate-specific membrane antigen, Prostate cancer, Hematotoxicity, Radioligand therapy

## Abstract

**Purpose:**

Hematotoxicity is a potentially dose-limiting adverse event in patients with metastasized castration-resistant prostate cancer (mCRPC) undergoing prostate-specific membrane antigen (PSMA)-directed radioligand therapy (RLT). We aimed to identify clinical or PSMA-targeted imaging-derived parameters to predict hematological adverse events at early and late stages in the treatment course.

**Methods:**

In 67 patients with mCRPC scheduled for ^177^Lu-PSMA-617 RLT, pretherapeutic osseous tumor volume (TV) from ^68^Ga-PSMA-11 PET/CT and laboratory values were assessed. We then tested the predictive capability of these parameters for early and late hematotoxicity (according to CTCAE vers. 5.0) after one cycle of RLT and in a subgroup of 32/67 (47.8%) patients after four cycles of RLT.

**Results:**

After one cycle, 10/67 (14.9%) patients developed leukocytopenia (lymphocytopenia, 39/67 [58.2%]; thrombocytopenia, 17/67 [25.4%]). A cut-off of 5.6 × 10^3^/mm^3^ for baseline leukocytes was defined by receiver operating characteristics (ROC) and separated between patients with and without leukocytopenia (*P* < 0.001). Baseline leukocyte count emerged as a stronger predictive factor in multivariate analysis (hazard ratio [HR], 33.94, *P* = 0.001) relative to osseous TV (HR, 14.24, *P* = 0.01). After four cycles, 4/32 (12.5%) developed leukocytopenia and the pretherapeutic leukocyte cut-off (HR, 9.97, *P* = 0.082) tended to predict leukocytopenia better than TV (HR, 8.37, *P* = 0.109). In addition, a cut-off of 1.33 × 10^3^/mm^3^ for baseline lymphocytes separated between patients with and without lymphocytopenia (*P* < 0.001), which was corroborated in multivariate analysis (HR, 21.39, *P* < 0.001 vs. TV, HR, 4.57, *P* = 0.03). After four cycles, 19/32 (59.4%) developed lymphocytopenia and the pretherapeutic cut-off for lymphocytes (HR, 46.76, *P* = 0.007) also demonstrated superior predictive performance for late lymphocytopenia (TV, HR, 5.15, *P* = 0.167). Moreover, a cut-off of 206 × 10^3^/mm^3^ for baseline platelets separated between patients with and without thrombocytopenia (*P* < 0.001) and also demonstrated superior predictive capability in multivariate analysis (HR, 115.02, *P* < 0.001 vs.TV, HR, 12.75, *P* = 0.025). After four cycles, 9/32 (28.1%) developed thrombocytopenia and the pretherapeutic cut-off for platelets (HR, 5.44, *P* = 0.048) was also superior for the occurrence of late thrombocytopenia (TV, HR, 1.44, *P* = 0.7).

**Conclusions:**

Pretherapeutic leukocyte, lymphocyte, and platelet levels themselves are strong predictors for early and late hematotoxicity under PSMA-directed RLT, and are better suited than PET-based osseous TV for this purpose.

## Introduction

As a type-2 transmembrane protein overexpressed on the surface of prostate and prostate cancer cells, the prostate-specific membrane antigen (PSMA) has emerged as a promising imaging target for men afflicted with prostate cancer (PC) [1, 2]. Thus, PSMA-targeted positron emission tomography (PET)/computed tomography (CT) has witnessed expanded use, mainly due to its strikingly higher accuracy in detecting sites of disease relative to conventional imaging [3]. In addition, increased PSMA expression visualized on ^68^Ga-PSMA-ligand PET can guide the referring treating physician towards radioligand therapy (RLT) with ^177^Lu-labeled equivalents [4–7]. Retrospective studies investigating PSMA-targeted RLT demonstrated high efficacy, even in patients with widespread metastatic disease [8]. The first prospective phase 2 study (LuPSMA trial) further corroborated these findings yielding a PSA decline of >50% in 57% of the enrolled subjects [9]. Further building on these encouraging results, the prospective VISION phase-3 trial will further elucidate the clinical value of PSMA RLT in metastasized castration-resistant prostate cancer (mCRPC) [10].

Although ^177^Lu-PSMA RLT has a high safety profile [11], it can be associated with hematological side effects, including leukocytopenia, lymphocytopenia, and thrombocytopenia [5, 8]. In this regard, reduced leukocyte counts occur in up to 40% of the patients during repeated cycles of RLT [8]. When Common Terminology Criteria for Adverse Events (CTCAE) are applied, more than 7% of patients demonstrate grade 3 or 4 adverse events [8]. Thus, the safety profile of RLT could be further increased if patients at risk for leukocytopenia or thrombocytopenia could be identified, preferably prior to treatment on-set. To date, no reliable parameter for predicting hematological adverse events has been established. For instance, it has been speculated that tumor volume (TV) in the skeleton may serve as a potential predictor for myelotoxicity, which could be explained by the cross-fire effect of beta irradiation within the bone marrow [12]. Thus, in the present study, we aimed to assess the predictive value of PET-derived osseous TV relative to a standard laboratory panel for CTCAE-defined leukocytopenia, lymphocytopenia, and thrombocytopenia at early and later stages in the treatment course.

## Material and methods

### Patient population

In this monocentric, retrospective study, 67 patients with mCRPC were included (Table 1). ^68^Ga-PSMA-11 PET/CT and ^177^Lu-PSMA-617 RLT were performed between June 2016 and January 2020. All patients demonstrated progressive disease under androgen deprivation therapy. The vast majority of patients did not longer respond to second-line antihormonal therapy including enzalutamide and abiraterone acetate, and to chemotherapy. We also performed an additional analysis in a subcohort of 32 patients receiving four cycles of RLT to investigate adverse events later in the treatment course. This retrospective study was approved by the institutional review board (No. 9182_BO_S_2020), compliant to the Declaration of Helsinki (“unproven interventions in clinical practice”) and the German Medicinal Products Act, AMG §13.2b. We administered ^177^Lu-PSMA-617 after obtaining written informed consent from each patient for both PSMA-targeted imaging and therapy as well as retrospective analysis. Parts of this cohort have also been investigated in [7].

**Table 1:** Patient characteristics

Parameter	Main cohort (*n* = 67)	Subcohort (*n* = 32)*	*P* value^+^
Age (years, mean ± SD)	72.43 ± 7.16	73.58 ± 7.05	0.348
Gleason score	8 (7–9)	8 (7–9)	0.675
Previous treatments (%)			
Radical prostatectomy	63	66	0.779
Primary radiation therapy	9	3	0.218
Salvage radiation therapy	57	59	0.805
Antihormonal treatment	100	100	1
Enzalutamide	64	60	0.648
Abiraterone acetate	61	66	0.674
Chemotherapy	81	78	0.777
Baseline laboratory values (median with interquartile range)			
Leukocyte count (×1000/mm^3^)	7.2 (5.55–8.25)	7.2 (4.78–8.03)	0.388
Lymphocyte count (×1000/mm^3^)	1.19 (0.74–1.6)	1.45 (1.1–1.64)	0.154
Platelets (×1000/mm^3^)	220 (193–272)	213 (193–256)	0.689
Hemoglobin (g/dl)	12 (10.9–13)	12.3 (11.8–13)	0.142
LDH (U/l)	269 (228–369.5)	259 (214–303)	0.268
AST (U/l)	26.5 (22–37)	25 (22–35.3)	0.846
eGFR (ml/min/1.73m^2^)	83 (63.25–89)	75.5 (63.25–86.5)	0.575
AP (U/l)	111 (71–242)	88 (68.5–207.5)	0.653
PSA (μg/l)	130.6 (28.3–429.3)	126.3 (38–627.8)	0.632
Osseous TV derived from pretherapeutic ^68^Ga-PSMA PET (cm^3^)	54.5 (2.58–228.88)	40.36 (0.81–207.26)	0.81

*AP* alkaline phosphatase, *AST* aspartate transaminase, *eGFR* estimated glomerular filtration rate, *LDH* lactate dehydrogenase, *PSA* prostate-specific antigen, *PSMA* prostate-specific membrane antigen, *RLT* radioligand therapy, *TV* tumor volume

*Subcohort with patients receiving 4 cycles of RLT

^+^*P* value of two-tailed Student’s *t* test, *P* values over 0.05 indicate similarity between the two cohorts

### Imaging procedure and volumetric assessment of osseous tumor burden

All studies were acquired using a dedicated PET/CT system (Biograph mCT 128 Flow; Siemens), equipped with an extended field-of-view PET component and a 128-slice spiral CT component, as previously described [13]. Patients received an intravenous injection of 105.1 ± 21.9 MBq of ^68^Ga-PSMA-11. Imaging started with a low-dose nonenhanced helical CT (120 kV, mA modulated, pitch of 1.2, reconstructed axial slice thickness of 5.0 mm) for attenuation correction. Whole-body PET images were subsequently acquired using continuous bed motion at a speed of 0.9 mm/s for chest and abdomen and 2.1 mm/s for legs at 1 h p.i.. All studies were reconstructed using Ultra HD, an iterative algorithm combined with time-of-flight and point-spread function information (Siemens Healthcare; 2 iterations, 21 subsets; matrix, 200; zoom, 1.0; Gaussian filter, 5.0). No contrast material was administered. PET images were analyzed using a commercial software package (syngo.via; V10B; Siemens Healthcare), allowing simultaneous and fused review of PET and CT data. In a consensus setting (LW, RAW), image interpretation to assess osseous TV was performed using PSMA-Reporting and Data System (RADS) version 1.0 [14]. Briefly, PSMA-RADS 1–3 categories classify rather benign lesions, whereas PSMA-RADS 4 and 5 lesions represent metastases attributable to PC and thus, the latter categories were included. For further details, refer to [14]. Volumetric parameters were calculated creating an isocontour volume of interest including all voxels above 45% of the maximum, as described in [13]. We then calculated osseous TV for all patients by summing up the volume of all PSMA-RADS 4 and 5 lesions located in the skeleton per patient.

### ^177^Lu-PSMA-617 RLT

A GMP-compliant preparation of the PSMA-targeting ligand ^177^Lu-PSMA-617 was performed as described previously [7]. Patients received 6.84 ± 0.75GBq of ^177^Lu-PSMA-617 intravenously. Treatment followed the national consensus recommendation for the use of PSMA RLT [15]. During treatment, patients underwent intravenous hydration with NaCl 0.9% (0.5 L before and 1 L after treatment). ^177^Lu-PSMA RLT was performed every 6–8 weeks and terminated when progressive disease or any major adverse events occurred.

### Assessment of laboratory values

Blood samples were collected prior to first RLT (cycle 1 day 1), during follow-up after 52 ± 11 days (cycle 2 day 1) and for the subcohort receiving 4 cycles of RLT after 222 ± 62 days (cycle 5 day 1). Blood collection was performed with di-potassium-ethylendiaminetetraacetic acid (EDTA) Monovette® tubes (Sarstedt, Nürnbrecht, Germany). Analysis of platelets, lymphocyte, and leukocyte counts were performed using impedance measurements to assess routine hematology. A Sysmex XN-10 analyzer (Sysmex Deutschland GmbH, Norderstedt, Germany) was used and operated according to manufacturers’ instructions and our in-house procedure guideline [16]. Standard quality assurance procedures were also routinely performed [16]. Hematotoxicity was defined according to CTCAE version 5.0 as leukocyte count under 3.6 × 10^3^/mm^3^, lymphocyte count under 1 × 10^3^/mm^3^, or platelets under 160 × 10^3^/mm^3^ [17]. In addition, estimated glomerular filtration rate (eGFR) was also calculated following the CKD-EPI equation [18]. In all subjects (100%), laboratory values at baseline and after one cycle of RLT were available. A subcohort of 32/67 (47.8%) patients was available for analysis after 4 cycles of RLT.

### Statistical analysis

Statistical analyses were performed using GraphPad Prism 9 (GraphPad Software, LCC) and SPSS Statistics 27 Inc. (IBM, Chicago). Two-sided Student’s *t* test was performed to compare two independent groups. Cut-offs for the prediction of adverse-events after one cycle of RLT were determined by ROC analysis using the Youden Index for optimization of sensitivity and specificity. We determined the relation between adverse hematological events and baseline laboratory values or TV using Fisher’s exact test. In addition, we performed univariate Kaplan-Meier analysis and nonparametric log-rank test utilizing the ROC-derived cut-offs to identify outcome differences between subgroups. Finally, multivariate Cox regression was performed to directly compare the predictive value of the most promising hematological parameter and TV for adverse events. In addition, the ROC-derived cut-offs established from analyzing adverse events after one cycle of RLT were re-investigated in the subcohort receiving four cycles. A *P* value <0.05 was considered statistically significant.

## Results

Exactly 55/67 (82.1%) of patients (Table 1) demonstrated metastatic disease in the skeleton. In total, 4111 bone lesions (PSMA-RADS 4, *n* = 1155; PSMA-RADS 5, *n* = 2956) were investigated and the median osseous TV was 54.5cm^3^ (IQR, 2.58–228.88cm^3^).

### Pretherapeutic leukocyte count is the strongest predictor for early and late leukocytopenia under RLT

**Fig. 1:**
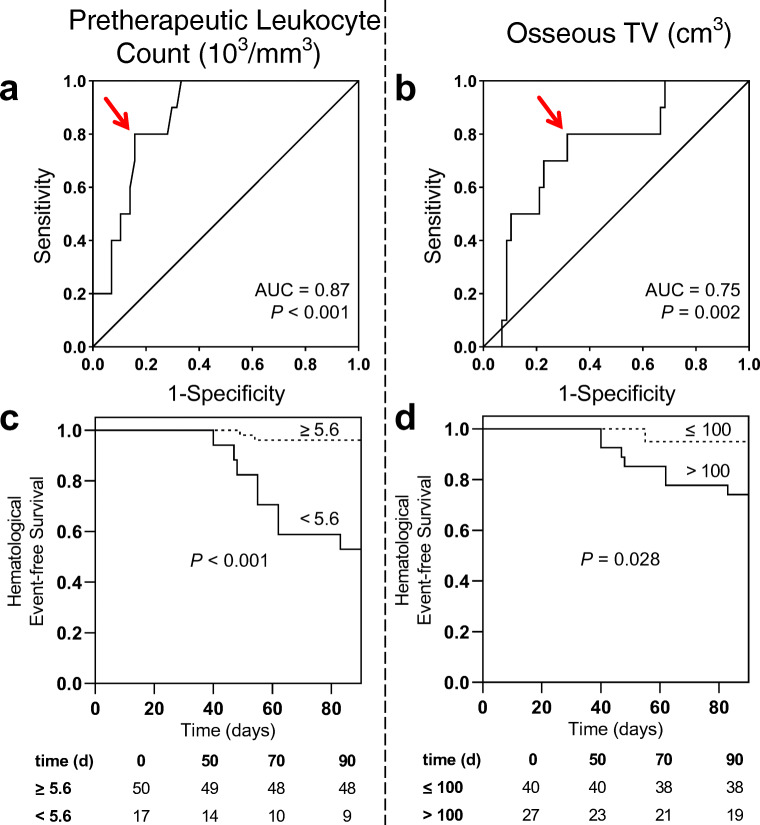
*Baseline leukocyte count and osseous tumor volume for prediction of leukocytopenia.* Receiver operating characteristics for the prediction of leukocytopenia for baseline leukocyte count (**a**) and osseous tumor volume (**b**). *Red arrows indicate optimal cut-offs with maximum sensitivity and specificity.* Kaplan-Meier curves for event-free survival of leukocytopenia for baseline leukocyte count (**c**) and osseous tumor volume (**d**) using ROC-derived cut-offs of 5.6 × 10^3^/mm^*3*^ for leukocyte count and 100cm^*3*^ for osseous tumor volume

After one cycle of RLT, leukocyte count declined from 7.13 ± 2.36 to 5.82 ± 2.17 × 10^3^/mm^3^ (*P* < 0.001); 10/67 (14.9%) patients developed leukocytopenia according to CTCAE (grade 1, 9; grade 2, 1). In receiver operating characteristics (Fig. 1a, b), baseline leukocytes demonstrated the highest accuracy in identifying subjects with and without leukocytopenia with an AUC of 0.87 (*P* < 0.001, best threshold, 5.6 × 10^3^/mm^3^), followed by osseous TV (AUC 0.75, *P* = 0.002, best threshold, 100cm^3^) and PSA (AUC 0.71, *P* = 0.02, best threshold, 260 μg/l). Neither LDH (AUC 0.68, *P* = 0.06, best threshold, 300 U/l), eGFR (AUC 0.64, *P* = 0.13, best threshold, 84 ml/min/1.73m^2^), AST (AUC 0.61, *P* = 0.23, best threshold, 25 U/l) nor AP (AUC 0.61, *P* = 0.3, best threshold, 303 U/l) were significantly associated. Baseline leukocyte count emerged as the strongest univariate predictor for leukocytopenia under RLT (OR, 21.33 [95% CI 3.88–117.39], *P* < 0.001), followed by osseous TV (OR, 8 [95% CI 1.55–41.43], *P* = 0.01; Table 2). In Kaplan-Meier analysis (Fig. 1c, d), baseline leukocyte count (*P* < 0.001) and osseous TV (*P* = 0.028) separated between patients with and without leukocytopenia under RLT. Baseline leukocyte count, however, emerged as a stronger predictive factor in multivariate analysis (HR, 33.94 [95% CI 4.48–257.51], *P* = 0.001) when compared to osseous TV (HR, 14.24 [95% CI 1.8–112.8], *P* = 0.01; Table 3).

**Table 2:** Univariate analysis of predictors for leukocytopenia under RLT

Parameter	OR	95% CI	*P* value
Leukocyte count	21.33	3.88 to 117.39	<0.001*
AST	3.72	0.73 to 19.12	0.166
eGFR	3.89	0.91 to 16.69	0.083
LDH	5.17	0.98 to 27.34	0.067
AP	5.88	1.38 to 25.01	0.022*
PSA	4.67	1.08 to 20.1	0.038*
Osseous tumor volume	8	1.55 to 41.43	0.011*

*CI* confidence Interval, *AP* alkaline phosphatase, *AST* aspartate transaminase, *eGFR* estimated glomerular filtration rate, *LDH* lactate dehydrogenase, *OR* odds ratio, *PSA* prostate-specific antigen

*Reached statistical significance

**Table 3:** Multivariate analysis of predictors for leukocytopenia under RLT

Parameter	After one cycle of RLT			After four cycles of RLT		
	HR	95% CI	*P* value	HR	95% CI	*P* value
Leukocyte count	33.94	4.48 to 257.51	0.001*	9.97	0.75 to 133.17	0.082
Osseous tumor volume	14.24	1.8 to 112.8	0.01*	8.37	0.62 to 112.84	0.109

*CI* confidence interval, *HR* hazard ratio, *RLT* radioligand therapy

*Reached statistical significance

**Fig. 2:**
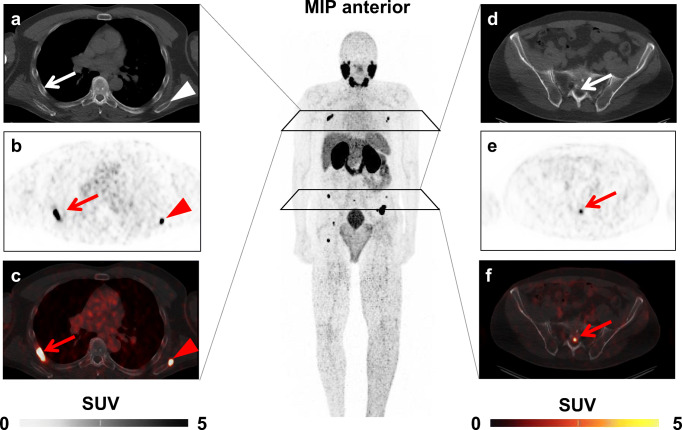
*Case example.* Baseline ^68^Ga-PSMA ligand positron emission tomography (PET)/computed tomography (CT) of a 59-year-old patient demonstrating several bone metastases, exemplified by PSMA-avid lesions in the sixth right rib (*arrow*) and left scapula (*arrowhead*) on **a** CT (without correlate), **b** PET, and **c** fused PET/CT images, and in the sacrum (*arrow*) (**d**–**f**). Baseline leukocyte count was 4.6 × 10^3^/mm^3^ which was below the receiver operating characteristics derived cut-off of 5.6 × 10^3^/mm^3^ (indicative of elevated risk of leukocytopenia). Osseous tumor volume (TV) was 12.01cm^3^, which was below the cut-off of 100cm^3^ and thus, PSMA-TV in the skeleton would suggest a reduced risk of hematological events. This patient, however, showed grade 1 leukocytopenia after one cycle of radioligand therapy. *MIP* maximum intensity projection, *SUV* standardized uptake value

After four cycles of RLT, leukocyte count declined from 6.71 ± 2.12 to 5.3 ± 1.56 × 10^3^/mm^3^ (*P* < 0.001); 4/32 (12.5%) developed leukocytopenia according to CTCAE (grade 1, 3; grade 2, 1). In a multivariate analysis using the previously established cut-offs, pretherapeutic leukocyte count (HR, 9.97 [95% CI 0.75–133.17], *P* = 0.082) tended to predict leukocytopenia better than osseous TV (HR, 8.37 [95% CI 0.62–112.84], *P* = 0.109) at later stage in the treatment course (Table 3). A case example is shown in Fig. 2**.**

### Pretherapeutic lymphocyte count is the strongest predictor for early and late lymphocytopenia under RLT

**Fig. 3:**
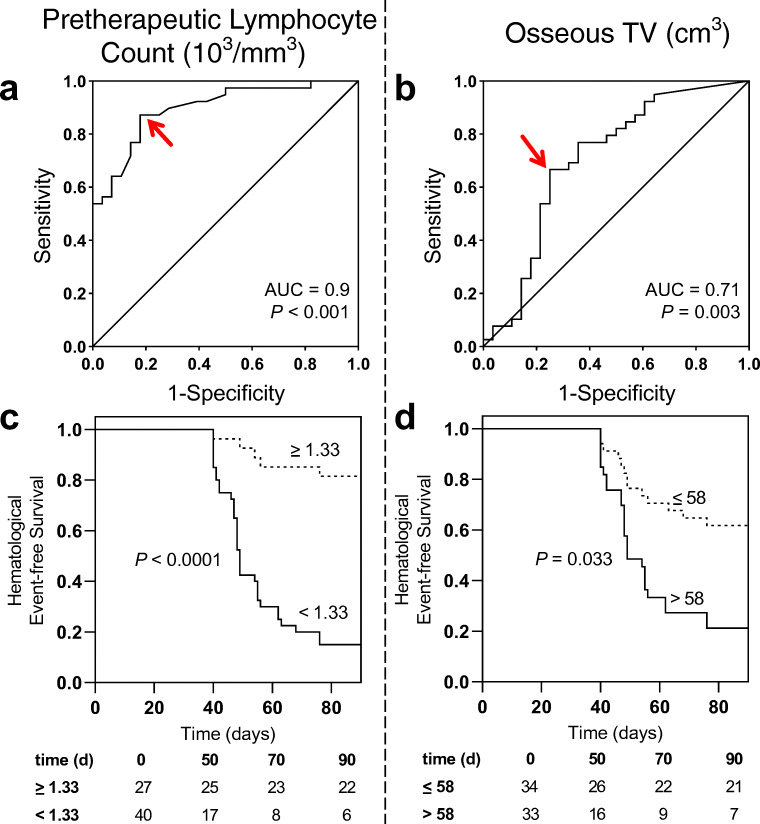
*Baseline lymphocyte count and osseous tumor volume for prediction of lymphocytopenia.* Receiver operating characteristics for the prediction of lymphocytopenia for baseline lymphocyte count (**a**) and osseous tumor volume (**b**). *Red arrows indicate optimal cut-offs with maximum sensitivity and specificity.* Kaplan-Meier curves for event-free survival of lymphocytopenia for baseline lymphocyte count (**c**) and osseous tumor volume (**d**) *using ROC-derived cut-offs of 1.33 × 10*^*3*^*/mm*^*3*^ *for lymphocyte count and 58cm*^*3*^ *for osseous tumor volume*

After one cycle of RLT, lymphocyte count declined from 1.21 ± 0.61 to 0.98 ± 0.53 × 10^3^/mm^3^ (*P* < 0.001); 39/67 (58.2%) developed lymphocytopenia according to CTCAE (grade 1, 14; grade 2, 14; grade 3, 10; grade 4, 1). In receiver operating characteristics (Fig. 3a, b), baseline lymphocytes demonstrated the highest accuracy in identifying subjects with and without lymphocytopenia with an AUC of 0.9 (*P* < 0.001, best threshold, 1.33 × 10^3^/mm^3^), followed by osseous TV (AUC 0.71, *P* = 0.003, best threshold, 58cm^3^) and PSA (AUC: 0.66, *P* = 0.024, best threshold, 30 μg/l). Neither AP (AUC 0.63, *P* = 0.074, best threshold, 155 U/l), LDH (AUC 0.6, *P* = 0.171, best threshold, 303 U/l), AST (AUC 0.57, *P* = 0.36, best threshold, 21 U/l) nor eGFR (AUC 0.52, *P* = 0.779, best threshold, 78 ml/min/1.73m^2^) reached significance. Baseline lymphocyte count emerged as the strongest univariate predictor for lymphocytopenia under RLT (OR, 24.93 [95% CI 6.78–91.7], *P* < 0.001), followed by osseous TV (OR, 6 [95% CI 2.03–17.74], *P* = 0.001; Table 4). In Kaplan-Meier analysis (Fig. 3c, d), baseline lymphocyte count (*P* < 0.0001) and osseous TV (*P* = 0.033) separated between patients with and without lymphocytopenia under RLT. Baseline lymphocyte count, however, emerged as a stronger predictive factor in multivariate analysis (HR, 21.39 [95% CI 5.44–84.12], *P* < 0.001) when compared to osseous TV (HR, 4.57 [95% CI 1.16–18.08], *P* = 0.03; Table 5).

**Table 4:** Univariate analysis of predictors for lymphocytopenia under RLT

Parameter	OR	95% CI	*P* value
Lymphocyte count	24.93	6.78 to 91.7	<0.001*
AST	4.03	1.09 to 14.84	0.057
eGFR	1.55	0.57 to 4.16	0.458
LDH	2.67	0.92 to 7.76	0.076
AP	2.64	0.93 to 7.49	0.079
PSA	5.89	1.78 to 19.51	0.004*
Osseous tumor volume	6	2.03 to 17.74	0.001*

*CI* confidence interval, *AP* alkaline phosphatase, *AST* aspartate transaminase, *eGFR* estimated glomerular filtration rate, *LDH* lactate dehydrogenase, *OR* odds ratio, *PSA* prostate-specific antigen

*Reached statistical significance

**Table 5:** Multivariate analysis of predictors for lymphocytopenia

Parameter	After one cycle of RLT			After four cycles of RLT		
	HR	95% CI	*P* value	HR	95% CI	*P* value
Lymphocyte count	21.39	5.44 to 84.12	<0.001*	46.76	2.89 to 757.98	0.007*
Osseous tumor volume	4.57	1.16 to 18.08	0.03*	5.15	0.5 to 52.69	0.167

*CI* confidence interval, *HR* hazard ratio, *RLT* radioligand therapy

*Reached statistical significance

**Fig. 4:**
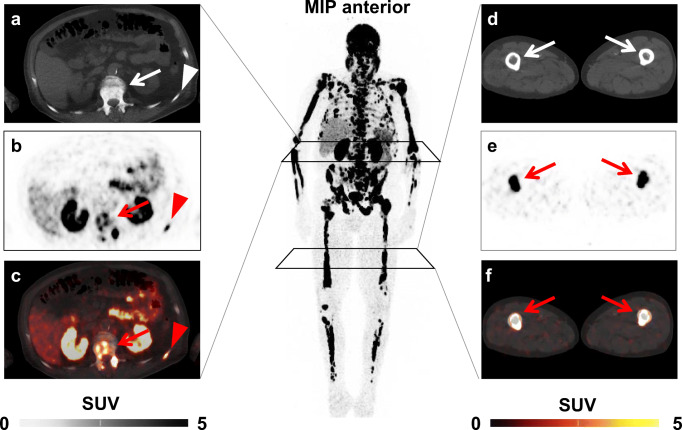
*Case example.* Baseline ^68^Ga-PSMA ligand positron emission tomography (PET)/computed tomography (CT) of an 83-year-old patient demonstrating extensive osseous tumor burden, exemplified by PSMA-avid sites of disease in the second lumbar vertebral body (*arrow*) and 11th left rib (*arrowhead*) on **a** CT, **b** PET, and **c** PET/CT. PSMA-avid lesions in both femurs (*arrows*) (**d**–**f**). Baseline lymphocytes were 1.6 × 10^3^/mm^3^, which was over the receiver operating characteristics derived cut-off of 1.33 × 10^3^/mm^3^ (indicative for no elevated risk of lymphocytopenia). Osseous tumor volume was 460.98cm^3^, which was above the cut-off of 58cm^3^ and thus, would suggest the occurrence of lymphocytopenia. This patient, however, showed no lymphocytopenia after one cycle of radioligand therapy. *MIP* maximum intensity projection, *SUV* standardized uptake value

After four cycles of RLT, lymphocyte count declined from 1.38 ± 0.48 to 0.89 ± 0.39 × 10^3^/mm^3^ (*P* < 0.001); 19/32 (59.4%) developed lymphocytopenia according to CTCAE (grade 1, 5; grade 2, 9; grade 3, 5). In a multivariate analysis using the previously established cut-offs, pretherapeutic lymphocyte count (HR, 46.76 [95% CI 2.89–757.98], *P* = 0.007) demonstrated superior predictive value for lymphocytopenia at late stage in the treatment course while osseous TV (HR, 5.15 [95% CI 0.5–52.69], *P* = 0.167) did not reach significance (Table 5). A case example is shown in Fig. 4.

### Pretherapeutic platelet count is the strongest predictor for early and late thrombocytopenia under RLT

**Fig. 5:**
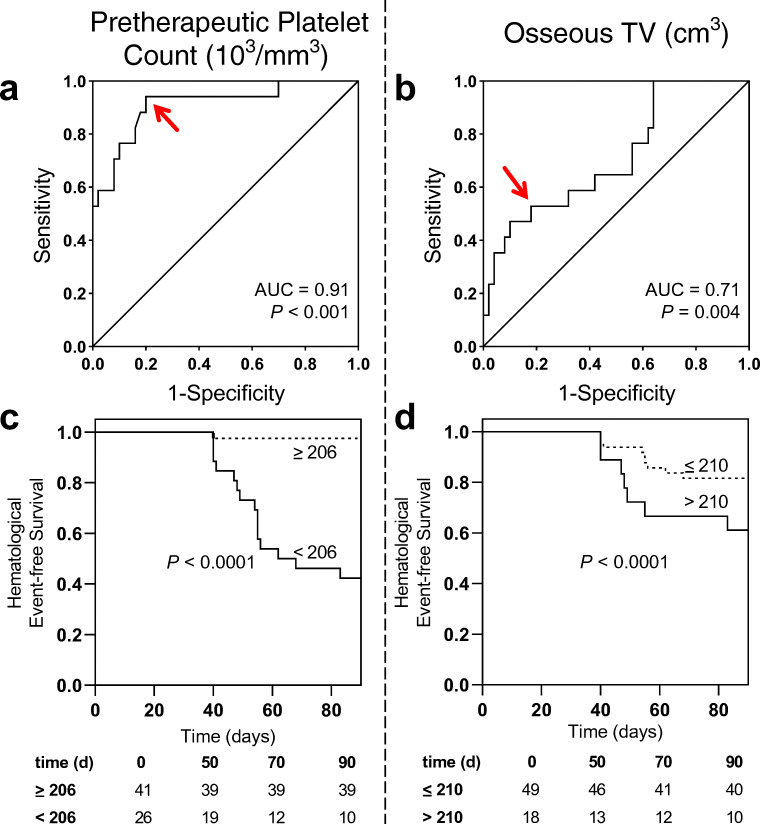
*Baseline platelet count and osseous tumor volume for prediction of thrombocytopenia.* Receiver operating characteristics for the prediction of thrombocytopenia for baseline platelets (**a**) and osseous tumor volume (**b**). *Red arrows indicate optimal cut-offs with maximum sensitivity and specificity.* Kaplan-Meier curves for event-free survival of thrombocytopenia for baseline platelets (**c**) and osseous tumor volume (**d**) *using ROC-derived cut-offs of 206 × 10*^*3*^*/mm*^*3*^ *for platelet count and 210cm*^*3*^ *for osseous tumor volume*

Platelets declined from 239 ± 82 to 218 ± 76 × 10^3^/mm^3^ (*P* = 0.008) after one cycle of RLT; 17/67 (25.4%) developed thrombocytopenia according to CTCAE (grade 1, 16; grade 2, 1). In receiver operating characteristics (Fig. 5a, b), baseline platelets demonstrated the highest accuracy in identifying subjects with and without thrombocytopenia with an AUC of 0.91 (*P* < 0.001, best threshold, 206 × 10^3^/mm^3^), followed by LDH (AUC 0.72, *P* = 0.003, best threshold, 300 U/l), osseous TV (AUC 0.71, *P* = 0.004, best threshold, 210cm^3^), AP (AUC 0.67, *P* = 0.02, best threshold, 280 U/l), and AST (AUC 0.661, *P* = 0.033, best threshold, 25 U/l). PSA (AUC 0.62, *P* = 0.167, best threshold, 260 μg/l) and eGFR (AUC 0.59, *P* = 0.231, best threshold, 77 ml/min/1.73m^2^) did not reach significance. Baseline platelets emerged as the strongest univariate predictor for thrombocytopenia under RLT (OR, 64 [95% CI 7.56–541.7, *P* < 0.001), followed by AP (OR, 6 [95% CI 1.69–21.26], *P* = 0.006), LDH (OR, 5.44 [95% CI 1.5–19.67], *P* = 0.009), AST (OR, 5.26 [95% CI 1.34–20.71], *P* = 0.013), and osseous TV (OR, 5.13 [95% CI 1.55–16.93], *P* = 0.01; Table 6). In Kaplan-Meier analysis (Fig. 5c, d), lower baseline platelets and higher osseous TV was associated with shorter event-free survival for thrombocytopenia (*P* < 0.0001). Baseline platelet counts, however, emerged as a stronger predictive factor in multivariate analysis (HR, 115.02 [95% CI 8.57–1543.11], *P* < 0.001; Table 7) when compared to osseous TV (HR, 12.75 [95% CI 1.38–118.01], *P* = 0.025).

**Table 6:** Univariate analysis of predictors for thrombocytopenia

Parameter	OR	95% CI	*P* value
Platelet count	64	7.56 to 541.7	<0.001*
AST	5.26	1.34 to 20.71	0.013*
eGFR	2.5	0.77 to 8.17	0.162
LDH	5.44	1.5 to 19.67	0.009*
AP	6	1.69 to 21.26	0.006*
PSA	3.04	0.98 to 9.35	0.082
Osseous tumor volume	5.13	1.55 to 16.93	0.01*

*CI* confidence interval, *AP* alkaline phosphatase, *AST* aspartate transaminase, *eGFR* estimated glomerular filtration rate, *LDH* lactate dehydrogenase, *OR* odds ratio, *PSA* prostate-specific antigen

*Reached statistical significance

**Table 7:** Multivariate analysis of predictors for thrombocytopenia

Parameter	After one cycle of RLT			After four cycles of RLT		
	HR	95% CI	*P* value	HR	95% CI	*P* value
Platelet count	115.02	8.57 to 1543.11	<0.001*	5.44	1.01 to 29.25	0.048*
Osseous tumor volume	12.75	1.38 to 118.01	0.025*	1.44	0.23 to 9.12	0.7

*CI* confidence interval, *HR* hazard ratio, *RLT* radioligand therapy

*Reached statistical significance

**Fig. 6:**
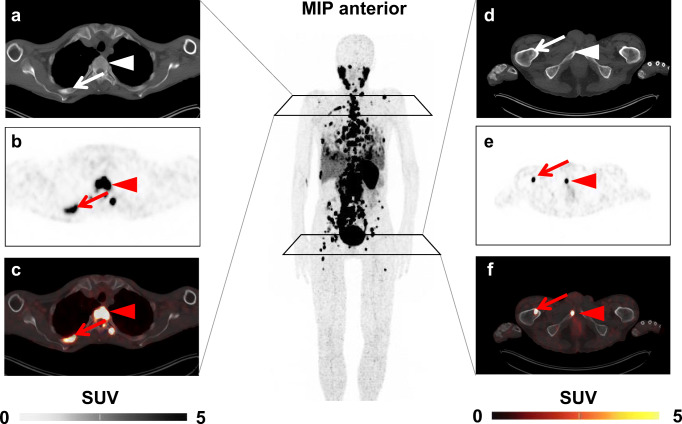
*Case example.* Baseline ^68^Ga-PSMA ligand positron emission tomography (PET)/computed tomography (CT) of a 67-year-old patient demonstrating multiple bone lesions, exemplified by PSMA-avid sites of disease in the fourth right rib (*arrow*) and the third thoracic vertebral body (*arrowhead*) on **a** CT, **b** PET, and **c** fused PET/CT. PSMA-avid lesion in the right femur (*arrow*) and pubic bone (*arrowhead*) (**d**–**f**). Baseline platelets were 190 × 10^3^/mm^3^, which was below the receiver operating characteristics derived cut-off of 206 × 10^3^/mm^3^ (indicative for elevated risk of thrombocytopenia). Osseous tumor volume was 72.97cm^3^, which was below the cut-off of 210cm^3^ and thus, would suggest no event. This patient, however, showed grade 1 thrombocytopenia with a decline of 41 × 10^3^/mm^3^ after one cycle of radioligand therapy. *MIP* maximum intensity projection, *SUV* standardized uptake value

After four cycles of RLT, platelets declined from 232.38 ± 71.83 to 198.84 ± 55.86 × 10^3^/mm^3^ (*P* < 0.003); 9/32 (28.1%) developed thrombocytopenia according to CTCAE (grade 1, 9). In a multivariate analysis, the superior predictive value of the previously established cut-off for pretherapeutic platelets (HR, 5.44 [95% CI 1.01–29.25], *P* = 0.048) was confirmed when compared to osseous TV (HR, 1.44 [95% CI 0.23–9.12], *P* = 0.7; Table 7). A case example is shown in Fig. 6.

## Discussion

In the present study enrolling a large cohort of mCRPC patients under RLT, baseline platelets, lymphocyte, and leukocyte counts demonstrated superior capability for predicting early hematological adverse event after one cycle of RLT relative to PET-based tumor burden in the skeleton. In addition, when re-investigating the established cut-offs for prediction of leuko-, lympho-, and thrombocytopenia after four treatment cycles, the superior predictive performance of baseline laboratory values was confirmed. Compared to a time-consuming manual segmentation assessing all osseous sites of disease throughout the entire body, pretherapeutic blood cell counts can be easily derived from a simple blood collection prior to treatment. Therefore, the herein presented cut-offs could be implemented in clinical routine to identify patients at risk for leuko-, lympho-, or thrombocytopenia early and late in the treatment course.

PSMA-targeted RLT is increasingly used, in particular for men afflicted with mCRPC, which have progressed under common first- and second-line therapeutic regimen [11]. Thus, the vast majority of these individuals have extensive tumor load on pretherapeutic PSMA-ligand PET/CT. Cross-fire effect of beta irradiation within the bone marrow has been advocated to cause hematological events including thrombocytopenia, lymphocytopenia, and leukocytopenia [12], in particular in subjects with multiple cycles of RLT [8] or widespread PSMA-avid disease in the skeleton [8]. Thus, one may speculate that osseous TV assessed from baseline PSMA-ligand PET/CT may serve as a suitable predictor for later hematological events. In our study, the predictive capability of tumor burden in the skeleton, however, did not outperform routine hematology prior to RLT, which demonstrated substantial higher HRs in multivariate analyses for leukocytopenia, lymphocytopenia, and thrombocytopenia after one cycle of RLT. These findings were further confirmed in mCRPC patients which had undergone four treatment cycles. Of note, a sophisticated approach of lesion detection attributable to PC has been applied, as only malignant PSMA-RADS 4 and 5 lesions have been segmented, while benign classified RADS 1-3D lesions have been excluded from further analysis [14]. As such, >4100 sites of disease in the skeleton were identified, but despite such a thorough analysis of osseous TV throughout the entire body, a simple blood collection was still superior in predicting adverse events occurring early and late in the treatment course, even in patients with massive skeletal burden. This might be explained by the fact that there is no close relationship between tumor load and bone marrow reserve, e.g., due to an expansion of active bone marrow in distal regions in patients suffering from massive skeletal burden, which could be explored performing bone marrow scintigraphy in future studies. Besides determining disease involvement in the skeleton on pretherapeutic PSMA-ligand PET, future studies investigating the absorbed dose on posttherapeutic whole-body scintigraphy may further increase predictive performance for hematological adverse events [19]. Posttherapeutic scintigraphy, however, is not available prior to RLT and reliable predictors for hematotoxicity should be accessible before administration of the therapeutic compound. Therefore, the herein presented cut-offs for pretherapeutic blood cell counts can help the referring physician in assessing the risk for hematological side effects prior to treatment on-set. Nonetheless, the decision to withhold RLT should not be exclusively based on such thresholds. Other factors may also play an important role, e.g., the individual quality of life, alternative treatment options, or previous biochemical response to RLT [11].

The herein presented data revealed that a substantial number of patients developed significant lymphocytopenia (grades 3 and 4, 16.4%) after one cycle of RLT, which may emphasize the relevance of hematological side effects under treatment. In this regard, the radiosensitivity of lymphocytes and their potential for biological dosimetry under RLT may also be subject of future studies [20]. However, similar to our study, previous reports showed that the frequency of thrombocytopenia and leukocytopenia in patients under RLT is rather low [5, 8, 15, 19]. As such, the reported mean changes of platelets and leukocyte counts after one cycle of RLT were both within the normal range for the total study population, although relevant hematotoxicity was seen in individual patients. The frequency of hematological side effects may be linked to the radionuclide used. ^177^Lu has a lower maximum ß-particle energy of 0.498 MeV relative to the radiometal ^90^Y as a pure ß-emitting radioisotope (2.28 MeV) [21]. This, however, also translates to shorter maximum penetration depth of the latter radionuclide of 1.7 mm (^90^Y, 11 mm) and thus, ^177^Lu may cause less cross-fire effect in the bone marrow leading to less thrombocytopenia and leukocytopenia during follow-up [21]. For instance, Rathke and co-workers reported on a higher frequency of hematological toxicity in 60% of the subjects when ^90^Y-PSMA-617 was administered. Although the investigated cohort was rather low (10 patients), hematological toxicity seemed to be increased in patients treated with ^90^Y-PSMA-targeted therapy when compared to ^177^Lu [22]. In this regard, Kurth et al. reported on bone marrow doses under ^90^Y-PSMA-617 which were fivefold higher relative to its ^177^Lu-labeled counterparts [23]. Thus, given a substantial proportion of patients that do not respond to ^177^Lu-PSMA [9], treatment with ^90^Y may serve as a suitable alternative in the future, in particular in men with extensive TV as larger lesions could benefit from the enhanced cross-fire effect [23]. Moreover, ongoing trials are also investigating combination treatments including RLT with nonsteroidal antiandrogens or chemotherapies [24]. Taken together, these novel endoradiotherapeutic approaches will have most likely similar or even higher rates of hematological events when compared to ^177^Lu-PSMA. Thus, future studies should also compare osseous TV to routine hematology to test whether a simple blood collection has also superior predictive performance in patients scheduled for ^90^Y-PSMA or ^177^Lu-PSMA in combination with other hematotoxic (chemo)therapies.

Some limitations should be acknowledged. First, a limited number of patients was enrolled and thus, our preliminary findings should be confirmed in a larger cohort, preferably in a prospective set-up. Second, although we were able to identify predictors for leuko-, lympho-, and thrombocytopenia, we were not able to evaluate such parameters for anemia. First, the vast majority of the investigated patients already developed anemia before RLT. In addition, hemoglobin did not significantly decline after one cycle of RLT (11.82 ± 1.52 vs. 11.65 ± 1.46; *P* = 0.09). Furthermore, potentially detrimental complications are often linked to leukopenia (e.g., sepsis) or thrombocytopenia (e.g., fatal bleeding), whereas RBCs can be easily transfused. Thus, we refrained from further analyses. Moreover, while our study mainly focused on short-term effects, predictors for long-term influence of RLT on hematology and bone marrow should be also assessed, even beyond four cycles of therapy. EANM procedure guidelines recommend time intervals of 6–8 weeks between repeated cycles of RLT to enable for recovery from potential myelosuppression [11]. Following these guidelines, we decided to assess follow-up laboratory values as late as possible, i.e., before initiating the next cycle of RLT. However, interim analyses, e.g., on a weekly basis after therapy, may be also of interest to assess potential recovery of leukocytes or platelets and to provide a more detailed time-course on the herein investigated hematological side effects under RLT. As alluded to earlier, bone marrow doses from posttherapeutic scintigraphy should also be determined to test whether such a sophisticated approach may be even more helpful in identifying high-risk individuals. Moreover, partial volume effects in investigated lesions in the skeleton on PSMA-ligand PET may further limit the value of the herein presented quantitative parameters derived from baseline PET. Furthermore, all osseous lesions independent of PSMA-RADS categories could also be investigated. The present approach of exclusively considering RADS 4 and 5 lesions, however, may represent a more standardized assessment, which will then allow for a better reproducibility of our findings, e.g., in a prospective setting.

## Conclusions

In the present study, enrolling a large cohort of mCRPC patients under PSMA-directed RLT, baseline platelet, leukocyte, and lymphocyte counts demonstrated superior capability for predicting thrombocytopenia, leukocytopenia, and lymphocytopenia early and later in the treatment course. Of note, standard blood values were better suited for this purpose when compared to a more time-consuming segmentation of the entire PET-based TV in the skeleton. As such, routine hematology prior to treatment should be evaluated to identify high-risk patients prone to early and late hematotoxicity after one or four cycles of PSMA-targeted therapy.
